# Effects of Different Recovery Modalities on Delayed Onset Muscle Soreness, Recovery Perceptions, and Performance Following a Bout of High-Intensity Functional Training

**DOI:** 10.3390/ijerph20043461

**Published:** 2023-02-16

**Authors:** Francine De Oliveira, Gabriel Andrade Paz, Victor Gonçalves Corrêa Neto, Renato Alvarenga, Silvio R. Marques Neto, Jeffrey M. Willardson, Humberto Miranda

**Affiliations:** 1 LADTEF—Performance, Training, and Physical Exercise Laboratory, Universidade Federal do Rio de Janeiro, Rio de Janeiro 21941-901, Brazil; 2 Programa de Pós-Graduação em Educação Física, Escola de Educação Física e Desportos, Universidade Federal do Rio de Janeiro, Rio de Janeiro 21941-901, Brazil; 3 School of Health Sciences, Gama e Souza University Center, Rio de Janeiro 22621-090, Brazil; 4 Biodesp Kinesiology Center of Performance, Rio de Janeiro 22790-704, Brazil; 5 Physical Education Graduate School, Estácio de Sá University (UNESA), Rio de Janeiro 20771-004, Brazil; 6 SALUS-Integrated Laboratory for Research in Exercise, Biomedicine and Public Health, Universidade Federal do Rio de Janeiro, Rio de Janeiro 21941-901, Brazil; 7 Physical Activity Sciences Graduate Program, Salgado de Oliveira University (UNIVERSO), Rio de Janeiro 24030-060, Brazil; 8 Health and Human Performance Department, Montana State University Billings, Billings, MT 59101, USA

**Keywords:** static stretching, foam rolling, training response, functional fitness

## Abstract

The purpose of this study was to investigate the effects of the foam rolling technique and static stretching on perceptual and neuromuscular parameters following a bout of high-intensity functional training (HIFT), which consisted of 100 pull-ups, 100 push-ups, 100 sit-ups, and 100 air squats (Angie benchmark) in recreationally trained men (*n* = 39). Following baseline measurements (Feeling Scale, Visual Analogue Scale, Total Quality Recovery, Sit-and-Reach, Countermovement Jump, and Change-of-Direction *t*-test), the volunteers performed a single bout of HIFT. At the end of the session, participants were randomly assigned to one of three distinct groups: control (CONT), foam rolling (FR), or static stretching (SS). At the 24 h time-point, a second experimental session was conducted to obtain the pos*t*-test values. The level of significance was set at *p* < 0.05. Regarding power performance, none of the three groups reached pretest levels at 24 h point of the intervention. However, the CONT group still showed a greater magnitude of effect at the 24 h time-point (ES = 0.51, *p* ≥ 0.05). Flexibility presented the same recovery pattern as power performance (post × 24 h CONT = ES = 0.28, FR = ES = 0.21, SS = ES = 0.19). At 24 h, all groups presented an impaired performance in the COD *t*-test (CONT = ES = 0.24, FR = ES = 0.65, SS = ES = 0.56 *p* ≥ 0.05). The FR protocol resulted in superior recovery perceptions (pre × 24 h TQR = ES = 0.32 *p* ≥ 0.05). The results of the present study indicate that the use of FR and SS exercises may not be indicated when aiming to restore neuromuscular performance following a single bout of HIFT. The use of the FR technique during the cooldown phase of a HIFT session may be helpful in improving an individual’s perception of recovery.

## 1. Introduction

High-intensity functional training (HIFT) can be described as a multimodal training routine primarily composed of functional movements performed at high exertion levels [1,2,3]. Previously, it has been proposed that functional movements involve the whole body, multi-segment, multiple planes of movement, and universal motor recruitment [3]. Most investigations conducted regarding the use of HIFT as a training modality use templates or benchmarks from CrossFit^®^. According to the brand, functional movements are performed in a wave of contraction from the core to an extremity which is directly related to motor control and postural stability concepts. Several positive adaptations have been observed with HIFT in a short time frame, such as improved body composition, resting heart rate, muscular endurance, strength, flexibility, bone mineral density, and skill-specific performance [3,4].

Despite being an efficient training modality, the absence of predefined resting periods, its circuit-style format, and the involvement of bodyweight or free-weight exercises may impose a considerable degree of physiological stress during each training session [5,6] that can promote delayed onset muscle soreness (DOMS) [7]. Therefore, an important aspect to consider is the recovery modality between HIIT sessions.

In this context, performance parameters have been used to assess fatigue and recovery noninvasively, such as the loss of muscle power measured through a countermovement jump (CMJ) as well as increased time for a change in direction test [8,9,10]. In addition, perceptual measurements such as well-being questionnaires and perceived exertion scales have been used to monitor fatigue, DOMS, and training-induced stress [11]. Proper recovery from a training protocol is imperative for amateur and professional athletes as well as physically active individuals. The technical demand imposed by the selected exercises during HIFT sessions, combined with high training volumes, metabolic demand, and perceived exertion, makes recovery modalities critical for this training mode. In this context, foam rolling (FR) has been considered a popular recovery modality. This self-massage technique seems useful when aiming to recover from EIMD and to promote gains in joint range of motion without detriments to strength, which is considered key in maintaining adequate performance between competitions and training sessions [12].

Foam rolling uses different tools, such as polypropylene rollers and balls, to apply a compressive force to soft tissue, but its mechanisms are a matter of constant debate [13]. Currently, an increased blood flow, the consequent greater removal of fatigue-inducing metabolites, and changes in thixotropic characteristics of the fascial tissue have been proposed as possible mechanisms [14]. Alternatively, static stretching (SS) has been traditionally prescribed following different modalities and intensities of exercise during a cooldown phase, even though recent evidence neither supports nor contradicts the use of postexercise stretching [15]. To the authors’ knowledge, no previous study assessed the effectiveness of FR and SS following a bout of HIFT on performance and subjective recovery perceptions. Thus, the purpose of this study was to investigate the effects of different recovery modalities on DOMS, recovery perceptions, and performance following a bout of high-intensity functional training in trained men. The authors hypothesized that the FR technique would provide greater benefits for the perceptual and neuromuscular recovery measures than the SS.

## 2. Materials and Methods

### 2.1. Experimental Design

This study had an experimental approach in which the sample was selected by convenience and used a predefined “benchmark” named “Angie” for a HIFT session which consisted of 100 pull-ups, 100 push-ups, 100 air squats, and 100 sit-ups performed as fast as possible [16]. One of the authors was responsible for scoring performance using a handheld counter. Participants were assessed before (“pre”), following (“post”), and 24 hours (“24 h”) following the training session. The recovery modalities (foam rolling, static stretching, or passive rest) were conducted immediately following the “post” measurements. Tests were all conducted in the same predefined order, and participants were allocated by simple random sampling. Height and body mass were recorded barefoot, and leg length was measured as the distance from the greater trochanter to the hallux with the subject laying on their back. Height at 90° knee flexion was defined as the vertical distance between the great trochanter and the ground in a seated position. Leg length and height at 90° were assessed wearing shoes. Tests included the “Feeling Scale” (FS), the pain “Visual Analogue Scale’ (VAS), and the Total Quality Recovery (TQR) scale as qualitative measurements; the “Sit and Reach” (SR), the Countermovement Jump (CMJ), and the change-of-direction *t*-test (COD *t*-test) as quantitative measurements.

Familiarization of the qualitative measurements was conducted online through video or voice call for a week before the first testing session, and familiarization of the quantitative measurements was conducted on the first day of testing consisting of two maximum “pretest” attempts in each of the evaluations after the warm-up with their respective resting interval. Following familiarization with the evaluations, testing would begin. Baseline assessments (“pre”) for FS, VAS, and TQR were taken prior to the warm-up protocol, which consisted of five minutes of running with change-of-direction sprints and five minutes of mobility drills. Following the warm-up, the SR, CMJ, and COD *t*-test were performed, and the HIFT session was conducted. All the evaluations were repeated immediately following the training session (“post”), and participants were randomly assigned to the recovery protocols. Participants returned 24 hours later (“24 h”) for retesting.

### 2.2. Sample and Ethical Procedures

Sample size calculation was conducted using GPower 3.1 software (Heinrich-Heine-Universität, Düsseldorf) considering an effect size *f* equal to 0.25, *alpha* equal to 0.05, and power equal to 0.80 reaching a total sample size of 36 individuals [5]. Thirty-nine participants were recruited for analysis considering the possibility of dropouts (age 29.2 ± 3.6 years; body mass: 84.4 ± 11.9 kg; height: 1.75 ± 0.06 m, and HIFT training experience of 3.1 ± 1.6 years). None of the participants dropped out of the investigation. Inclusion criteria included (a) male participants; (b) between 25 and 35 years old; (c) at least one year of experience in HIFT; (d) regularly practicing the modality at least twice a week. Exclusion criteria consisted of (a) any musculoskeletal injury that led to training absence for eight days or more in the six months prior to testing; (b) use of anabolic steroids. 

Exclusion criteria were adopted during an anamnesis. Participants were instructed to maintain their regular dietary and sleep regimens. However, they were requested to refrain from all other recovery modalities that could blunt the results of the present investigation during the conduction of the study. In addition, participants refrained from any other kind of physical activity during the proposed timeframe. All participants were informed of the benefits and risks of the investigation prior to signing an institutionally approved informed consent document to participate in this study. This study was conducted according to the Declaration of Helsinki, and the Institutional Review Board of the corresponding university approved the experimental protocol (45373121.5.0000.5257).

### 2.3. FS, VAS, and TQR Testing Procedures

The FS is an instrument composed of eleven answering possibilities to the question “How are you feeling right now?” to evaluate the affective response to exercise [17]. VAS was used to assess DOMS perception in general. To standardize the instructions given to the participants and, consequently, the interpretation of the results, the extreme values of the scale were described to the participants, with “0” being the absence of pain and “10” being the greatest possible pain [9].

The TQR is a scale structured according to the Borg Scale and was initially applied so that the participants could describe their general recovery perception following a HIFT session [9]. For the (“post”) and (“24 h”) time-points, participants considered their perception of recovery three minutes following the training session alone and 24 h following the combination of the training session and their respective recovery modality [9].

### 2.4. Neuromuscular Testing Procedures

#### 2.4.1. The Sit-and-Reach (SR)

This test was conducted to evaluate lumbar and hamstrings flexibility and was performed according to procedures previously described elsewhere. During testing, subjects remained seated on the floor with extended knees and slightly separated legs with their feet pressed against the board. Arms were extended forward with the hands placed palms down on the upper surface of the scale. In this position, the subject bobs forward four times and holds the position of maximum reach on the fourth count. Two trials were conducted with a 30 s rest interval between trials. The mean value was used for statistical analysis [9].

#### 2.4.2. Countermovement Jump (CMJ)

Data collection regarding vertical jump performance was conducted using the MyJump App, previously validated for IOS cameras [9,17,18]. Participants started in a standing, neutral position with arms placed on their hips to avoid the contribution of the upper limbs to the jumping performance. During the execution of the jump, the knees could flex freely to perform the highest jump possible. Immediately following landing, the knees could slightly flex again. Two attempts were performed with a one-minute rest interval between them. If one of the attempts was not in accordance with the instructions given by the evaluators, another attempt was performed [9,17,18]. The mean value was used for statistical analysis [9].

#### 2.4.3. Change of Direction (COD) *t*-Test

Two testing attempts with a one-minute rest interval between them were conducted. Attempts were invalidated if the subject did not perform the test as instructed. When permitted, participants sprinted forward 9.14 m to touch the first cone. They then side-shuffled 4.57 m to the left and touched the second cone. Next, they side-shuffled 9.14 m to the right and touched a third cone, then 4.57 m to the left back to the point where the first cone was, touching it again. Finally, participants backpedaled 9.14 m. There was no permission to look back or sideways. Submaximal and maximal “pretest” attempts were performed to minimize the “learning effect” [19,20]. The test was conducted according to a previously outlined protocol [9,18], and the fastest trial was used for statistical analysis.

#### 2.4.4. Foam Rolling Protocol

Participants performed a 45 s bout of foam rolling followed by a 15 s rest twice, alternating left and right sides over the following muscle areas: hamstrings, gluteus, quadriceps, pectoralis, and latissimus dorsi. A total of 20 min of self-massage was completed [21]. The grid foam roller type was chosen to exert more pressure on the tissue [22]. 

#### 2.4.5. Static Stretching (SS)

Participants performed a 45 s bout of static stretching followed by a 15 s rest twice, alternating left and right sides over the following muscle areas: hamstrings, gluteus, quadriceps, pectoralis, and latissimus dorsi. A total of 20 min of static stretching was completed. Participants stretched to the point of mild discomfort [23].

#### 2.4.6. Passive Rest (Control Group)

Participants were instructed to sit quietly on a bench for 20 min.

#### 2.4.7. Training Load

To ensure that the groups had enough homogeneity for comparisons, internal training load in arbitrary units was calculated through the session rating perceived exertion method (RPE × session duration in minutes) (sRPE) [24]. Perceived exertion was assessed at the 30 min point after exercise to control for the influence of the high muscular exertion required by the session. Currently, the sRPE is the recommended method to monitor training loads in HIFT [24].

### 2.5. Statistical Analysis

Data were entered into IBM SPSS Statistics 20 software for analysis (SPSS, Chicago, III). Normality tests were conducted on all the quantitative variables. The assumption of normality was not rejected through the Shapiro–Wilk test, and histogram analysis confirmed the normality of the data. A 3 × 3 (group × time) repeated-measures analysis of variance (ANOVA) with Bonferroni´s post hoc adjustment for multiple comparisons was used. Mean and standard deviation values were used to describe normally distributed data. For the qualitative variables (FS, VAS, TQR), a Friedman test was conducted to assess differences between groups and treatments.

When Friedman´s test presented a statistical difference, pairwise comparisons adjusted for Bonferroni were automatically conducted. The median and interquartile range (IQR) was used to describe qualitative and nonparametric data. In addition, to confirm training load homogeneity, groups were considered independent of each other, and a Kruskal–Wallis test by rank was conducted after the typically assumed data was rejected [25]. The level of significance was set at 5% (*p* ≤ 0.05), and effect sizes were used for the interpretation of results. Cohen´s *d* was used for parametric data (≥0.2 small, ≥0.5 medium, and ≥0.8 large), and Cohen´s guidelines for “*r*” were also used for nonparametric data (the small effect is 0.1, the medium effect is 0.3, and the large effect is 0.5) [26].

## 3. Results

### 3.1. Training Load

There was no significant difference in training load (sRPE) between groups, *p* = 0.431; chi-square = 1.684, df = 2 arbitrary units CONT = 182 (70); FR = 175 (92); SS = 154 (65). 

#### 3.1.1. Feeling Scale, Visual Analogue Scale, Total Quality Recovery

Outcomes for FS, VAS, and TQR are presented as medians and interquartile ranges in Table 1. Effect sizes are displayed in Table 2. For the FS, the intra protocol analysis presented a significant difference between the “pre” and “post” time-points for the FR group (*p* = 0.043). Individuals allocated to the FR group had a worse affective response to the proposed training session, but this difference was not relevant to the point of causing a statistically significant difference between groups. Additionally, for the “SS” group, the Friedman test showed a significant difference between time-points (*p* = 0.049), but this difference was diluted following adjustment for pairwise comparisons. For the DOMS perception, a statistically significant difference was found between “pre” and “24 h” time-points for the “SS” group (ES: 0.18).

#### 3.1.2. The Sit-and-Reach, Countermovement Jump, and Change-of-Direction *t*-Test

Outcomes for SR, CMJ, and COD *t*-test are presented as means and standard deviations in Table 3. Effect sizes are displayed in Table 2. For the SR, no statistically significant differences were found between groups for any time-points (*p* = 0.420). The results of the ANOVA presented a significant effect of time (*p* = −0.001). Post hoc Bonferroni analysis showed statistically significant differences between the “pre” versus “post” (*p* = 0.002) and “pre” versus “24 h” (*p* = 0.005) time-points. Group * Time interaction showed differences for the ‘FR” group between the “post” versus “24 h” (*p* = 0.02 ES = 0.21), and for the “SS” group between the “pre” versus “post” (*p* = 0.003 ES = −0.38), and “post” versus “24 h” (*p* = 0.018 ES = 0.19) time-points. For the CMJ, no statistically significant differences were found between groups for any time-points (*p* = 0.755). The results of the ANOVA presented a significant effect of time (*p* = −0.001). Post hoc Bonferroni analysis showed statistically significant differences between the “pre” versus “post” (*p* = 0.001) and “post” versus “24 h” (*p* = 0.041) time-points. 

Group*Time interaction showed differences for the “CONT” group between the “pre” versus “post” (*p* = 0.023 ES = 0.90), for the “SS” group between the “pre” versus “post” (*p* = 0.023 ES = 0.31), and “post” versus “24 h” (*p* = 0.007 ES = 0.22) time-points. For the COD-T test, no statistically significant differences were found between groups for any time-points (*p* = 0.887). The results of the ANOVA presented a significant effect of time (*p* = 0.005). Post hoc Bonferroni analysis showed statistically significant differences between the “post” versus “24 h” (*p* = 0.011) time-points. Group*Time interaction showed significant differences for the “SS” group between moments “post” versus “24 h” (*p* = 0.014 ES = 0.56) time-points.

## 4. Discussion

Power performance measured through the CMJ presented the same recovery pattern in all three groups, which may indicate that recovery might be more dependent on the time frame rather than the recovery modality used. It is important to highlight that none of the three groups reached pretest levels 24 hours following the HIFT training session, irrespective of the recovery modality. The time course of recovery following exercise-induced muscle damage (EIMD) depends on various factors such as duration and intensity of the proposed exercise, muscle length, and muscle groups involved during the activity, which influences the extent of the initial muscle damage. Even though DOMS cannot be proposed as a direct marker of EIMD, it is considered a common symptom of muscle damage [27]. 

However, the CONT group showed a greater magnitude of effect 24 h postintervention (ES = 0.51) versus both experimental groups (FR = 0.29, SS = 0.22). FR was shown to acutely worsen fatigue indices (less resistance to fatigue) when the pace was not controlled [28,29], which raises the hypothesis of a possible deleterious effect of FR on power performance that may persist over an unknown time frame. Even though some studies propose using a metronome for pacing control (the control of movement velocity), this procedure tends to reduce the external validity of the investigation [28,29]. 

Previous research observed an acute deleterious effect of SS on ankle force sense acuity. This may consequently impair motor balance and control, which is critical for the adequate performance of functional activities [30]. In addition, persistent deleterious effects have been previously observed following bouts of SS on horizontal power and sprint performance versus dynamic stretching and control conditions [31]. It is possible that in the present study, the SS modality may have compromised the effectiveness of the stretch-shortening cycle by decreasing active musculotendinous stiffness and limiting the amount of elastic energy that could be stored and reused [31]. Furthermore, a possible reduction in neural drive to the muscle may be responsible for the observed force loss after the acute performance of prolonged duration (>60 s) static stretching; however, it is not clear if there is a persistent effect preventing motoneurons from discharging at their maximal firing rates [32]. Numerous mechanical and neural factors have been proposed as potential mechanisms for performance decrements after using different SS protocols, such as the capacity to alter the musculotendinous unit length, thus decreasing passive tension. Increased muscle compliance added to nociceptors’ excitability, and inhibition of proprioceptors, such as the Golgi tendon organs, reduce the activation of alpha motoneurons [33]. A reduction in muscle tone through less activation of muscle spindles also reduces the ability to generate force through the elastic component.

Twenty-four hours following testing, the three groups presented an impaired performance on the COD *t*-test (post × 24 h CONT = 0.24, FR = 0.65, SS = 0.56) that may be related to the presence of DOMS based on the visual analog scale (CONT = 0.43 FR = 0.34 SS = 0.41) [34,35]. In addition, as seen in the CMJ performance, the FR and the SS groups presented worse performance versus the CONT group 24 h postintervention, considering the magnitude of differences between time-points. It should be noted that power determinants have been associated with the change of direction performance in different samples, such as male adolescent basketball players and female soccer players [12,36]. The importance of vertical power expression in the change of direction performance may arise from the large forces applied to the ground when initiating a change of direction maneuver before a rapid hip extension, and the amount of acceleration that is part of the COD *t*-test may rely more heavily on important characteristics for linear speed as the stretch-shortening cycle [12,36].

The fact that the proposed HIFT session did not promote a deleterious effect on the ability to change direction and showed a possible improvement in performance in all three groups (CONT = 0.16, FR = 0.48, SS = 0.67) may be explained by a motor-learning effect even though a submaximal and two maximal attempts were conducted aiming to minimize this event [19,37]. This suggests that a greater number of maximal attempts should be performed before testing. In the present study, flexibility was assessed using the “sit-and-reach” presenting small changes from the second to the third time-points in all groups, showing the same recovery pattern as the CMJ performance (post × 24 h CONT = 0.28, FR = 0.21, SS = 0.19). The absence of statistically significant differences between groups regarding flexibility has been shown in a previous study conducted by Rey et al. [9] that used the same time frame and testing procedure following an intense bout of soccer. However, it is in contrast with the results found by MacDonald et al. [38], whose experimental design evaluated the recovery process for up to 72 h after a training session designed to exacerbate DOMS through a protocol that consisted of 10 sets of 10 maximum repetitions in a squat exercise. Conflicting results may emerge from different evaluation methods, as MacDonald et al. [38] combined a manual and an electronic goniometer to assess the quadriceps’ passive range of motion and hamstrings passive and dynamic range of motion. 

The authors highlight that the protocol proposed by MacDonald et al. [38] uses the FR technique for 72 h without performing any other exercise session in between, which normally does not represent the reality of most athletes and recreationally trained individuals. In addition, the use of the FR protocol caused a greater decrease in muscle contractile properties, such as the rate of force development, which may indicate that the use of the FR technique exacerbated EIMD, and perhaps corroborate the hypothesis of a possible deleterious after of FR on performance. Previous research compared the effects of active recovery (leg pedaling), low-frequency surface neuromuscular electrical stimulation, and passive recovery (control group) in a crossover design of performance, markers of physiological recovery, and perceptual measures. According to their results, HIFT practitioners can reach a similar recovery with any of the three modalities provided by their study [39]. This corroborates the findings of the present study, although there is a clear discrepancy regarding the experimental design and methods proposed. It should be noted that the study conducted by Martínez-Gómez et al. [39] performed recovery modalities only to the lower limbs, whereas the present study applied a whole-body intervention that may trigger a systemic response. 

Perceptual measures have been used to monitor training load in different sports settings [40,41]. In the present study, the FR technique seemed to provide better recovery perception measured through the “total quality recovery” (pre × 24 h TQR = 0.32). The possibility of a greater effect size should be considered if the intervention is conducted over a longer time frame. These results are in accordance with most of the previous research regarding the use of FR as a recovery tool since its benefits have been vastly associated with perceptual measurements [21].

DOMS seems to reach its peak 24–72 h postexercise. Considering that HIFT practitioners may perform the activity daily, a full investigation in a 72 h protocol is recommended. Regarding the use of SS, a recent meta-analysis has considered data as scarce, heterogeneous, and not supportive of existing guidelines that reinforce the use of static stretching as a recovery modality, making it difficult to support or contradict the use of SS as a postexercise recovery tool [15]. Still, the group performing SS as a modality showed a better magnitude of recovery represented by their pain perception (pre × 24 h FR = 0.38 SS = 0.44) and their affective response (pre × 24 h FR = 0.08 SS = 0.17) that may be related with the sum of the relaxation caused by the stretching protocol, which is contrary to the perception of pain that can be caused by the FR grid type, especially in individuals who are not accustomed to the self-massage technique. 

Preceding research classified HIFT sessions as a training concept derived from the combination of high-intensity interval training and traditional resistance training [2]. Compared with vigorous intensity resistance training, a 12-week high-intensity interval training protocol provided neurotrophic factors such as neurotrophin-3 and a trend towards increasing serum dopamine concentrations [42]. It should be highlighted that even though the present study investigates an acute experimental session, the central purpose is to observe the use of different recovery modalities, and this implies that this type of training session will be part of an individual’s training routine. Dopamine is considered key in many physiological functions, including motor control and aspects related to the modulation of mood and affective states. Additionally, dopamine plays a modulative role in the basal ganglia, with an important role in movement pattern formation and motivation [43], influencing athletes’ drive to exercise. In this scenario, elevated dopamine concentrations in the brain can drive athletes towards greater effort and motivate consistency [44]. Future research regarding dopamine expression during HIFT sessions should be conducted. 

The proposed HIFT session primarily consisted of upper body exercises, while the proposed neuromuscular tests were lower body. The recovery process involved several systems, and the fatigue imposed by the training session caused changes in several body structures, including the central nervous system (CNS). It is suggested that central fatigue promotes an inability of the CNS to optimally conduct the skeletal and muscular system, which highlights a more global approach to the recovery process [45]. Furthermore, the reduced ability of the CNS to appropriately activate nonexercised limbs has already been described and displays a crossover effect of fatigue [46]. Nevertheless, it should be pointed out that investigations regarding a crossover effect of fatigue have been mostly conducted on unilateral exercises aiming to observe a possible effect on the contralateral limb or between different activities, such as a reduction in manual tasks after an intense cognitive assignment [47]. More research is needed regarding a possible crossover effect between body segments. 

The present study had some limitations. During HIFT sessions, participants were instructed to perform “as fast as possible” or “as many as possible”, and resting periods were not defined, which may have exposed participants to different amounts and variations in the physiological stimulus. However, the lack of rigorous control of training variables guarantees greater ecological validity and maintains the main characteristics of this type of training. Second, participants were not highly familiarized with the foam rolling technique, which may have imposed greater effort and discomfort since the pain threshold might be lower in certain individuals causing the pressure of foam rolling to be highly painful. Lastly, the sample was composed only of male participants with extensive HIFT experience, which prevents the extrapolation of results for novice and female individuals. 

## 5. Conclusions

In summary, the results of the present investigation indicate that the use of foam rolling and static stretching exercises may not be indicated when aiming to restore neuromuscular performance following a single bout of HIFT. However, the study shows that using the FR technique during the cooldown phase of a HIFT session may be useful for improving an individual´s perception of recovery, while using the SS may provoke a greater effective response. Therefore, considering the recovery from an acute HIFT session, neither of the proposed modalities seems more efficient than passive recovery as participants appeared to attain similar short-term recovery. 

## Figures and Tables

**Table 1:** Outcomes of the FS, VAS, and TQR presented as median and interquartile range.

FS	“Pre”	“Post”	“24 h”	*p*-Value Intraprotocol
Control	3 (2)	1 (5)	3 (4)	*p* = 0.198
Foam Rolling	4 (2)	2 (3) *	3 (3)	*p* = 0.043 *
Static Stretching	3 (3)	1 (2)	3 (5)	*p* = 0.049 ^#^
*p*-value interprotocol	*p* = 0.153	*p* = 0.565	*p* = 0.368	
VAS	“pre”	“post”	“24 h”	*p*-value intraprotocol
Control	2 (5)	0.00 (3)	5 (5)	*p*= 0.086
Foam Rolling	2 (4)	2 (4)	6 (7)	*p*= 0.098
Static Stretching	0.00 (3)	0.00 (5)	4 (5) ^+^	*p* = 0.006 ^+^
*p*-value interprotocol	*p* = 0.215	*p* = 0.442	*p* = 0.458	
TQR	“pre”	“post”	“24 h”	*p*-value intraprotocol
Control	15 (3)	12 (4) ^$^	15 (4) ^$^	*p* = 0.001 ^$^
Foam Rolling	17 (4)	13 (4)	15 (4)	*p* = 0.050 ^#^
Static Stretching	15 (3)	13 (4) ^&^	15 (4)	*p* = 0.003 ^&^
*p*-value interprotocol	*p* = 0.133	*p* = 0.010 **	*p* = 0.934	

^#^ Diluted difference after adjusting for pairwise comparisons; * Statistically significant differences between “pre” and “post” time-points; ^+^ Statistically significant difference between “pre” and “24 h” time-points; ^$^ Statistically significant difference between “pre” versus “post” and “post” versus “24 h”; ^&^ statistically significant difference between “pre” versus “post”; ** Statistically significant difference between groups “FR” and “control”. FS—Feeling Scale; VAS—Visual Analog Scale; TQR—Total Quality Recovery.

**Table 2:** Effect sizes.

	Control			Foam Rolling			Static Stretching		
	pre × post	pre × 24 h	post × 24 h	pre × post	pre × 24 h	post × 24 h	pre × post	pre × 24 h	post × 24 h
SR (cm)	0.32 *	0.05	0.28 *	0.15	0.05	0.21 *	0.38 *	0.18	0.19 *
CMJ (cm)	0.90 *	0.34 *	0.51 *	0.37 *	0.08	0.29 *	0.31 *	0.09	0.22 *
COD (sec)	0.16	0.07	0.24 *	0.48 *	0.15	0.65 *	0.67 *	0.20 *	0.56 *
FS	0.35 ^#^	0.071	0.28 ^#^	0.44 ^#^	0.08	0.35 ^#^	0.33 ^#^	0.17 ^#^	0.35 ^#^
VAS	0.34 ^#^	0.22 ^#^	0.43 ^#^	0.17 ^#^	0.38 ^#^	0.34 ^#^	0.03	0.44 ^#^	0.41 ^#^
TQR	0.55 ^#^	0.017	0.57 ^#^	0.37 ^#^	0.32 ^#^	0.05	0.57 ^#^	0.17 ^#^	0.39 ^#^

* (≥0.2 small, ≥0.5 medium e ≥ 0.8 large) ^#^ small effect 0.1, medium effect 0.3, large effect 0.5. SR—Sit-and-Reach; CMJ—Countermovement Jump; COD—Change-of-Direction *t*-test; FS—Feeling Scale; VAS—Visual Analog Scale; TQR—Total Quality Recovery.

**Table 3:** Outcomes of the SR, CMJ, and COD presented as mean and standard deviation.

SR (cm)	“Pre”	“Post”	“24 h”	*p*-Value Intraprotocol
Control	25.98 ± 4.91	24.30 ± 5.32	25.71 ± 4.48	
Foam Rolling	26.82 ± 8.77	25.51 ± 8.17	27.30 ± 8.64	*p* = −0.001 *
Static Stretching	30.63 ± 7.32	27.86 ± 7.22	29.30 ± 7.19	
*p*-value interprotocol		*p* = 0.420		
CMJ (cm)	“pre”	“post”	“24 h”	*p*-value intraprotocol
Control	34.68 ± 2.44	32.42 ± 2.47	33.86 ± 2.72	
Foam Rolling	35.77 ± 5.52	33.66 ± 5.78	35.31 ± 5.29	*p* = −0.001 ^#^
Static Stretching	36.02 ± 7.78	33.70 ± 6.81	35.30 ± 7.17	
*p*-value interprotocol		*p* = 0.755		
COD (sec)	“pre”	“post”	“24 h”	*p*-value intraprotocol
Control	12.22 ± 0.89	12.37 ± 0.90	12.15 ± 0.91	
Foam Rolling	12.12 ± 0.85	12.65 ± 1.32	12.00 ± 0.67	*p* = −0.005 ^+^
Static Stretching	11.97 ± 1.53	13.03 ± 1.59	12.25 ± 1.19	
*p*-value interprotocol		*p* = 0.887		

* statistically significant differences for “FR” between “post” and “24 h” time-points (*p* ≤ 0.05), for “SS” between “pre” versus “post” time-points (*p* ≤ 0.05), and “post” versus “24 h” (*p* ≤ 0.05); ^#^ Statistically significant differences for “control” between “pre” and “post” time-points (*p* ≤ 0.05) and for “SS” between “pre” versus “post” time-points (*p* ≤ 0.05) as well as “post” versus “24 h” (*p* ≤ 0.05); ^+^ Statistically significant differences for “SS” between “post” and “24” time-points (*p* ≤ 0.05). SR—Sit-and-Reach; CMJ—Countermovement Jump; COD—Change-of-Direction *t*-test.

## Data Availability

Available through the corresponding author by request.
